# Generative AI and LLMs for Critical Infrastructure Protection: Evaluation Benchmarks, Agentic AI, Challenges, and Opportunities

**DOI:** 10.3390/s25061666

**Published:** 2025-03-07

**Authors:** Yagmur Yigit, Mohamed Amine Ferrag, Mohamed C. Ghanem, Iqbal H. Sarker, Leandros A. Maglaras, Christos Chrysoulas, Naghmeh Moradpoor, Norbert Tihanyi, Helge Janicke

**Affiliations:** 1 School of Computing, Engineering and the Built Environment, Edinburgh Napier University, 10 Colinton Road, Edinburgh EH10 5DT, UK; yagmur.yigit@napier.ac.uk (Y.Y.); n.moradpoor@napier.ac.uk (N.M.); 2 Department of Computer Science, Guelma University, Guelma 24000, Algeria; 3 Cybersecurity Institute, University of Liverpool, Liverpool L69 7ZX, UK; mcghanem@liverpool.ac.uk; 4 Cyber Security Research Centre, London Metropolitan University, London N7 8DB, UK; 5 Centre for Securing Digital Futures, Edith Cowan University, Perth, WA 6027, Australia; m.sarker@ecu.edu.au (I.H.S.); h.janicke@ecu.edu.au (H.J.); 6 School of Computer Science, De Montfort University, Gateway House, Leicester LE1 9BH, UK; leandros.maglaras2@dmu.ac.uk; 7 Department of Digital Media and Communication, Ionian University, Antonis Tritsis Ave, Argostoli, Kefalonia, 28100 Argostoli, Greece; 8 School of Mathematical & Computer Sciences, Heriot Watt University, Edinburgh EH14 4AS, UK; c.chrysoulas@hw.ac.uk; 9 Technology Innovation Institute (TII), Abu Dhabi P.O. Box 9639, United Arab Emirates; norbert.tihanyi@tii.ae

**Keywords:** critical national infrastructure, critical infrastructure protection, security, reliability

## Abstract

Critical National Infrastructures (CNIs)—including energy grids, water systems, transportation networks, and communication frameworks—are essential to modern society yet face escalating cybersecurity threats. This review paper comprehensively analyzes AI-driven approaches for Critical Infrastructure Protection (CIP). We begin by examining the reliability of CNIs and introduce established benchmarks for evaluating Large Language Models (LLMs) within cybersecurity contexts. Next, we explore core cybersecurity issues, focusing on trust, privacy, resilience, and securability in these vital systems. Building on this foundation, we assess the role of Generative AI and LLMs in enhancing CIP and present insights on applying Agentic AI for proactive defense mechanisms. Finally, we outline future directions to guide the integration of advanced AI methodologies into protecting critical infrastructures. Our paper provides a strategic roadmap for researchers and practitioners committed to fortifying national infrastructures against emerging cyber threats through this synthesis of current challenges, benchmarking strategies, and innovative AI applications.

## 1. Introduction

**Figure 1:**
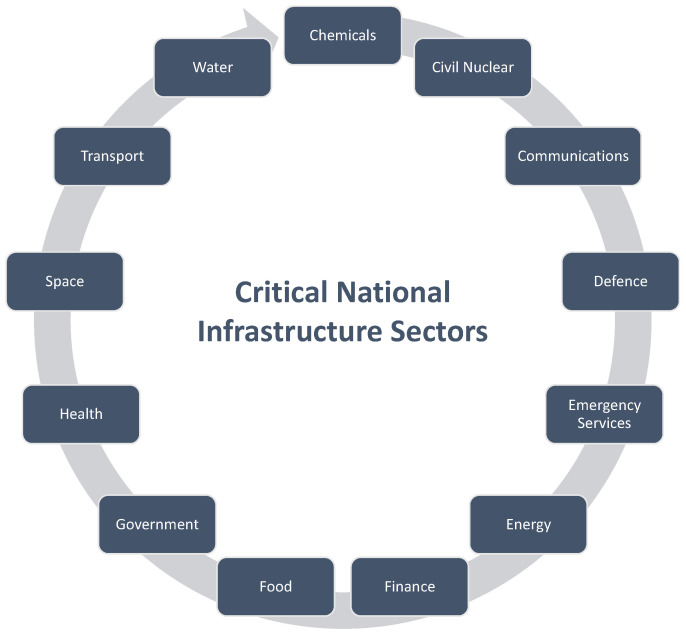
The critical national infrastructure sectors.

The critical national infrastructure (CNI) consists of the resources of a nation that are essential to the smooth operation of its economy and society. CNI encompasses the essential facilities, systems, sites, information, people, networks, and processes a country relies on for its daily operations and overall functioning. This includes crucial services as well as certain functions, sites, and organizations that, while not essential for daily operations, require protection due to their potential risk to public safety, such as civil nuclear and chemical sites. The thirteen national infrastructure sectors in the UK are chemicals, civil nuclear, communications, defence, emergency services, energy, finance, food, government, health, space, transport, and water, as shown in Figure 1. Some sectors, like emergency services, further break down into sub-sectors, such as police, ambulance, fire services, and coast guard [1].

In 2024, the frequency of global cyber attacks surged, with organizations experiencing an average of 1308 attacks per week in the first quarter [2]. This marks a 28% increase from the last quarter of 2023 and a 5% rise compared to the same period last year [3]. As these attacks become more common, the financial impact is also growing. The cybercrime losses are projected to skyrocket from $9.22 trillion in 2024 to $13.82 trillion by 2028 [4]. Moreover, high-impact attacks on critical infrastructure have increased by 140% [5]. A recent report highlights that over 150 industrial operations in sectors such as process manufacturing and critical industrial infrastructures faced cyber attacks with physical consequences in 2022. These incidents have grown 2.4 times from the previous year, and at this growth rate, up to 15,000 industrial sites could face shutdowns due to cyber attacks within the next five years.

The emergence of Industry 4.0 [6], along with the increased connectivity of devices associated with CNI and the integration of traditional computer networks, has expanded the attack surface of these critical assets. The attacks on CNI have been an ongoing issue for decades, and they appear to be growing in number, frequency, and impact. For example, in December 2015 [7], the world witnessed the first power outage caused by a cyber-attack. This attack, which began with a phishing attack, resulted from the BlackEnergy malware, a Trojan used for conducting distributed denial-of-service (DDoS) attacks, cyber espionage, and information destruction. It targeted utility companies in Ukraine, leaving hundreds without electricity for six hours. Moreover, cyberattacks have targeted water companies for twenty years [8]. For instance, in 2019 [9], a water-distribution company in Kansas (USA) experienced an attack by a former employee who gained remote control of the company’s information system and proceeded to tamper with the drinking water-treatment process. Furthermore, in 2021 [10], there were attacks on water-treatment infrastructure in Norway by ransomware named Ryuk. The hackers aimed to profit significantly by encrypting the company’s files and demanding a ransom. Additionally, APT34 [11] serves as an example of Advanced Persistent Threats (APTs), as identified by FireEye researchers in 2017. APT34 specifically targeted government organizations and financial, energy, chemical, and telecommunications companies in the Middle East. Furthermore, APT28 [12], a Russian group known as Fancy Bear, Pawn Storm, and Sednit, is another example of APTs targeting CNI. It was identified by Trend Micro in 2014 and conducted attacks against military and government targets in Ukraine and Georgia, as well as NATO organizations and US defence contractors.

Criminals and state-sponsored hackers are increasingly targeting CNI to disrupt society. They are probing for vulnerabilities, gathering intelligence, and exploiting individuals and systems for financial gain. Consequently, it is only a matter of time before a specific CNI becomes a direct target. The expectation that Industrial Control Systems (ICSs) and CNI are completely secure, isolated, and immune to attacks is no longer valid. No industry or organization can consider itself completely safe. Table 1 shows some significant cyberattacks targeting critical national infrastructure sectors since 2022, with losses of more than a million dollars [13].

It is crucial to protect CNI and ensure its reliability and cybersecurity because nations depend on its operation and consistency. Any disturbance to their operations could potentially devastate physical security, national security, economic wealth, safety, and health. This includes household and business destruction that may result in evacuations, business closures, financial losses, deaths, health hazards, and environmental impacts. To provide cybersecurity and reliability for CNIs, there are various efforts that both governments and agencies can employ, including:

- Implementing an all-hazards approach to risk management, considering cyber and physical threats to critical infrastructure integrity.
- Integrating Incident Response (IR) strategies with Business Continuity Planning (BCP) to ensure seamless continuity of operations during and after security incidents.
- Adopting a consequence-management approach to manage critical infrastructure failures’ immediate and long-term impacts, including economic, societal, and environmental consequences.
- Regularly assessing the security status of CNIs and conducting penetration testing to identify vulnerabilities and weaknesses.
- Employing robust security-mitigation measures such as intrusion-detection systems, cryptography methods, firewalls, anti-virus software, and emerging security technologies like blockchain, Artificial Intelligence (AI), and machine learning.
- Establishing and enforcing policies for maintaining and updating software and hardware periodically to mitigate vulnerabilities arising from outdated systems.
- Providing comprehensive cybersecurity training to staff to enhance awareness and preparedness against cyber threats.
- Enforcing robust cybersecurity policies and operating procedures, ensuring compliance with regulatory frameworks and industry standards.
- Encouraging international cooperation and coordination to address cross-border cyber threats effectively, including information sharing and joint response efforts.
- Collaborating with industry experts and sharing threat intelligence to stay ahead of emerging cyber threats and vulnerabilities.

Businesses can safeguard and preserve their critical infrastructure while guaranteeing that users will always have access to required services by putting these measures into practice. However, the tension between the requirement for information exchange and regulation and compliance must be acknowledged. Regulations are essential for establishing guidelines and guaranteeing accountability, but if they are burdensome, organizations are reluctant to report due to potential fines for law-breaking. Dealing with the cybersecurity challenges that CNIs encounter requires an open and cooperative culture, particularly in sectors where private businesses are common. Encouraging information exchange while maintaining regulatory control is necessary to achieve this. The National Institute of Standards and Technology (NIST) provides a comprehensive Risk-Management Framework (RMF) for protecting critical infrastructure systems against cyber threats. The NIST cybersecurity framework is widely used for risk assessment, incident response, and threat mitigation in CNI protection [14]. Similarly, ISO 27001 outlines globally accepted best practices for information security management, ensuring secure data handling, encryption, and compliance with regulatory standards [15].

There is great potential for Critical Infrastructure Protection (CIP) when cutting-edge technologies like Large Language Models (LLMs) and Generative AI are integrated. However, there are still many hurdles to overcome to close the gap between theoretical advancements and practical applications. To do so, the difficulties and potential paths forward for protecting critical infrastructure systems need to be thoroughly examined. This paper is a comprehensive review of existing cybersecurity threats to critical infrastructure, regulations, and security standards. We synthesize and evaluate existing research on security challenges, best practices, and emerging technologies such as Generative AI and LLMs. Our goal is to provide a structured analysis of existing work, highlight key challenges, and discuss future research directions in CIP. We provide an in-depth investigation of the co-analysis of safety and security, emphasizing the links between these fields and offering innovative integration techniques. Furthermore, we describe a comprehensive method for utilizing Generative AI and LLMs for CIP, provide an example lifecycle and discuss particular applications across multiple critical infrastructure industries. Finally, we suggest future paths to enhance critical infrastructure security and resilience. Searches were conducted using JSTOR (jstor.org) and Google Scholar using a combination of search methodologies. All papers were required to have been published in reputable peer reviewed journals no earlier than 2020. Some exceptions were made to this criterion if the papers provided a notable contribution to the field or were unique in the points raised. Having selected a list of potential papers these were then short-listed based on relevance to the specific topics selected, credibility of the publication, citation count, any novel or interesting approaches to the issues and a range of authors from different geographies to try and obtain a more balanced, global view of the issues. Inevitably, some papers were weighted towards specific areas, such as reliability, LLM and CNI, technical challenges or information security issues. This provided challenges for paper selection and review due to the broad range of areas covered. However, this article aims to provide a baseline assessment of the selected topics and should be read in the context of a foundation for further research.

Given the increasing complexity and interconnectivity of CNI systems, ensuring their reliability is crucial for maintaining operational continuity. Section 2 explores reliability-assessment techniques that help mitigate disruptions in CNI systems. Section 3 presents datasets used for benchmarking LLMs in cybersecurity. An overview of the cybersecurity threats to critical infrastructure networks and their operations is provided in Section 4. Section 5 delves into trust, privacy, and resilience requirements specific to CIP. Section 6 explores the interplay between safety and security and presents recent research advancements. In Section 7, we delve into the practical applications of Generative AI and LLMs for enhancing critical infrastructure resilience and security. Section 8 presents how Agentic AI can proactively mitigate operational risks and ensure system resiliency in complex environments. Section 9 discusses future directions for CIP. Lastly, Section 10 summarizes the key findings and outlines potential advancements for future research and innovation in CIP.

## 2. Reliability of Critical National Infrastructures

The reliability of CNIs is vital for critical networks and systems that operate normally since infrastructures support numerous businesses, including energy, transportation, telecommunications, and maritime ports. These industries offer critical services for a country’s population’s safety and well-being [16,17]. Reliability refers to the ability of these systems to do their responsibilities within set parameters and time restrictions [18]. A system is considered reliable if it meets the requirements of the application and has a high probability of successful operation over a specific period of time. Building a reliable system involves understanding each component’s overall dependability and how it interacts with other systems.

Installing security measures and offering reliable services to clients are the main responsibilities of the CNI. These components have the ability to recognize disturbances such as mistakes or cyberattacks that disrupt operations and respond accordingly [19]. Using statistical techniques, the CNI reliability examination analyzes the behaviour of the system and identifies any issues [20]. This section covers the most widely used techniques for evaluating system reliability, such as Monte Carlo simulation methodologies, Weibull analysis, and Markov Chains. In terms of system reliability, these are the best and most commonly used methods for defect analysis and system performance forecasts.

### 2.1. Weibull Analysis

The Weibull distribution methodology is the most efficient method for making inferences from failure data in components and systems. This method performs better even with small sample sizes than Poisson or binomial distribution methods [21]. It is beneficial since collecting large failure samples would be very expensive and dangerous.

The Weibull analysis is a practical tool for modelling system behaviour with regard to reliability. Predictive reliability analysis has the advantage of being able to simulate several data sources. The approach most frequently employed is the two-parameter Weibull distribution, which is comprised of the scale parameter α and the shape parameter β. These components must be thoroughly understood in order to comprehend the failure process of a system. The failure rate function, average lifespan, reliability function, and likelihood of failure at any given time may be easier to compute thanks to this type of analysis [21].

Understanding the probability density function is necessary in order to calculate the predicted rate of failures over time. This function’s expression is as follows:

(1) $$f\left(t;\alpha ,\beta \right)=\frac{\beta }{\alpha }{\left(\frac{t}{\alpha }\right)}^{\beta -1}e^{-{\left(\frac{t}{\alpha }\right)}^{\beta }}$$

The likelihood that a failure will occur by a given time *t* may be found using the cumulative distribution function. As demonstrated by:

(2) $$F\left(t;\alpha ,\beta \right)=1-e^{-{\left(\frac{t}{\alpha }\right)}^{\beta }}$$

The failure rate function calculates the imminent failure risk, assuming the object has survived till time *t*. It is described as

(3) $$\lambda \left(t;\alpha ,\beta \right)=\frac{f(t;\alpha ,\beta )}{1-F(t;\alpha ,\beta )}=\frac{\beta }{\alpha }{\left(\frac{t}{\alpha }\right)}^{\beta -1}$$

Failure intensity is mostly determined by the reliability function, which expresses the probability of surviving until at least time *t* [22]. It gives a direct measure of survival, which is a supplement to the probability density function and is represented as:

(4) $$R\left(t;\alpha ,\beta \right)=e^{-{\left(\frac{t}{\alpha }\right)}^{\beta }}$$

The Weibull analysis relies heavily on the interaction between the shape (β) and scale (α) parameters. Comprehending these parameters is imperative for precise reliability approximations, hence facilitating the refinement of maintenance tactics and product blueprints for amplified system effectiveness and dependability. The shape parameter β determines the failure rate’s trend over time, which indicates whether it rises, falls, or stays constant [23]. On the other hand, the failure data’s spread is impacted by the scale parameter α, which modifies the distribution’s time axis [24]. When these data are combined, it is possible to describe failure mechanisms precisely. It simplifies the process of building focused maintenance and replacement programs that boost system reliability.

Weibull analysis has been widely used for predicting transformer lifespan and grid component reliability. For instance, in power grid management, Weibull analysis is applied to assess the lifespan and failure probability of key components such as transformers and circuit breakers. This helps in scheduling predictive maintenance and preventing unexpected failures in energy-distribution systems.

### 2.2. Markov Chains

Markov Chains offer a mathematical framework for modelling and assessing the dependability of complexly interconnected and state-transitioning stochastic systems. Using Markov Chains, the reliability analysis approach measures the likelihood of system states over time while taking into consideration all potential states of operation, failure, and repair procedures. This method makes it possible to evaluate the performance of the system and find reliability indices that are useful for operations and maintenance plans.

A detailed examination of system reliability is achieved through a series of steps in the Markov Chain reliability analysis [25]. These operations consist of:

- Identifying every potential state of the system, including failure and normal states, to create a complete state space.
- Creating a transition probability matrix that shows the possibility of a state change at a given moment. This matrix is the foundation of Markov Chain analysis.
- Indication of the rates of transition between the states, including the rates of failure and repair. For continuous-time Markov Chains, it is usually assumed to follow exponential distributions.
- Determination of the long-term behaviour of the system by calculating the steady-state probability of each system state. This involves solving the balance equations derived from the transition probability matrix.
- Employing steady-state probabilities to determine key reliability metrics such as mean time to repair (MTTR), system availability, and mean time to failure (MTTF).

The state space *S* represents all possible states of the system, including operational and failure states.

(5) $$S=\{s_{0},s_{1},s_{2},\ldots ,s_{n}\}$$

**Figure 2:**
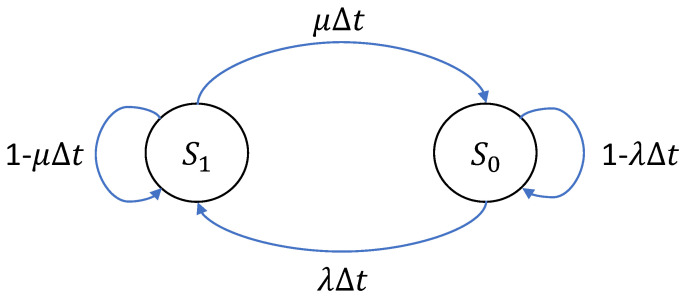
The representation of the transition of a two-state system.

The two-state system graph can be shown in Figure 2. In this diagram, $s_{0}$ represent normal state, while $s_{1}$ shows faulty state. The failure and repair rates are shown by λ and μ, respectively. The time interval Δt depicts a brief time in Figure 2 [25].

Markov Chains predict future states from existing situations. This is highly beneficial for investigating the dependability of CNI. The definition of the transition probability matrix *P* is given by $P_{ij}$, which denotes the likelihood of a single time step transfer from state $s_{i}$ to state $s_{j}$.

(6) $$P=\left[\begin{array}{cccc} P_{s_{1}1} & P_{s_{1}2} & \cdots & P_{s_{1}n}\\ P_{s_{2}1} & P_{s_{2}2} & \cdots & P_{s_{2}n}\\ \vdots & \vdots & \ddots & \vdots \\ P_{s_{n}1} & P_{s_{n}2} & \cdots & P_{s_{n}n} \end{array}\right]$$

The steady-state probabilities π are calculated by decoding πP=π with the condition $\sum_{s_{i}=1}^{n}\pi s_{i}=1$ [25]. These probabilities reflect the system’s long-term behaviour, indicating the likelihood of being in each state after a large number of transitions.

Key reliability metrics such as MTTF and system availability can be derived from the steady-state probabilities [26]. For a system with states categorized into operational and failure states, MTTF can be estimated as follows [27]:

(7) $$MTTF=\sum_{s_{i}\in \mathrm{Operational}\;\mathrm{States}}\frac{1}{\lambda s_{i}}$$

where $\lambda s_{i}$ is the failure rate of state $s_{i}$.

As the likelihood of the system being in an operational state at time *t* equals the total of the probabilities of all operational states, it is possible to calculate the reliability R(t) of the system [28].

(8) $$R\left(t\right)=\sum_{\mathrm{operational}\;\mathrm{states}\;i}\pi_{i}\left(t\right)$$

System availability A(t), considering both operational and repair states, is calculated as [25]:

(9) $$A\left(t\right)=\frac{\mu }{\lambda +\mu }$$

Assuming a simple two-state model. The transition probabilities are significantly influenced by the failure (λ) and repair (μ) rates of the elements, directly affecting the system’s reliability.

Several factors influence the reliability analysis utilizing Markov Chains, including λ, μ, *P*, and *S* [25]. The initial probability distribution among the states might affect how the system behaves in the short term and how well maintenance processes work. The likelihood of state changes affects the system’s overall performance and its capacity to sustain operational states over time. Increased failure rates make a failure scenario more likely, which lowers the system’s overall reliability. Increased repair rates enhance reliability and availability by allowing the system to keep running and learn from its errors.

The reliability analysis is also impacted by the relationships between the system’s numerous components because they alter the probability of a transition. Modelling these interactions consistently is necessary to achieve stable reliability values. A comprehensive and advanced method of comprehending a system’s behavior throughout time is provided by the model. Transition probabilities, steady-state probabilities, and failure rates combined offer a thorough method for predicting system performance and indicating possible areas for development.

Markov Chain models have been successfully applied in the management of water-distribution networks, where they predict the transition states of pipeline degradation and leak failures. Additionally, in cyber-physical security, Markov-based models assist in intrusion detection by modelling state transitions between normal operation and cyberattack scenarios.

### 2.3. Monte Carlo Simulation

The Monte Carlo simulation technique is becoming increasingly necessary in terms of reliability. It provides a dependable means of assessing the performance and reliability of complex systems. In many situations, the behaviour of complex systems can be predicted by statistical modelling and random sampling. This helps experts estimate the likelihood of system faults and identify important areas for improvement. That makes it an invaluable technique for assessing risk [29]. Utilizing probability distributions to depict the uncertainty in system performance and component dependability, this reliability analysis essentially models the system’s behaviour under various scenarios. By simulating numerous situations in which each component may fail following its failure distribution and seeing the system’s response to these failures, the reliability of a system is determined.

There are multiple important steps in the Monte Carlo Simulation process [20,30,31]:

- *Definition of a System Parameter:* Set up initial system parameters (*P*), such as operating conditions (μ), failure rates (λ), and repair rates.
- *Setup of the Simulation Model:* Create a model, S(t), that represents the system’s operational states over time, with S(t)=1 representing normal operation and S(t)=0 representing failure.
- *Requirements for Failure:* Define performance thresholds as the basis for failure criteria and designate the system as failed when its performance (*P*) falls below a given threshold ($P_{th}$).
- *Stochastic Sampling:* For every parameter, perform a random sampling from the corresponding probability distributions; for example, sample $t_{fail}$ for failure rates from Exp(λ).
- *Iteration and Statistical Analysis:* Conduct multiple simulation iterations (*N*) to observe various outcomes, calculating the system reliability (*R*) and time to failure (TTF) as follows:
  (10) $$R=\frac{1}{N}\sum_{i=1}^{N}S_{i}\left(t\right),$$
  where $S_{i}\left(t\right)$ represents the system state in the *i*-th iteration at time *t*.
- *Analysis of Results:* Estimate MTTF and reliability over the specified period with:
  (11) $$MTTF=\frac{\sum_{i=1}^{N}TTF_{i}}{N},$$
  where $TTF_{i}$ is the time to failure in the *i*-th iteration.

The failure rate (λ), system architecture, performance thresholds ($P_{th}$), and operational environment in Monte Carlo Simulations for reliability analysis interplay significantly [29,31]. An increase in λ typically reduces *R* and MTTF, indicating lower system reliability. Adds complexity to S(t) modelling, where redundancy can enhance *R* but also introduces additional variables to the simulation. Modifying $P_{th}$ alters the failure criteria, affecting *R* and MTTF calculations. Variations in operational conditions can impact λ and μ, thereby influencing *R* and MTTF.

The Monte Carlo simulation technique is highly practical in investigating the impact of diverse risk factors on CNI and estimating the effectiveness of different mitigation tactics. It comprehensively understands potential vulnerabilities and resilience strategies, considering various inputs and outcomes. It provides insights into how different factors contribute to system reliability, guiding design and maintenance strategies decision-making. Monte Carlo simulations are widely used in smart grid security to estimate the impact of cyberattacks on energy distribution. By simulating attack scenarios, system operators can predict vulnerabilities and optimize defensive strategies to enhance grid resilience.

Ensuring the reliability of CNI requires advanced risk-assessment techniques. However, with the growing role of AI in cybersecurity, evaluating these AI models becomes equally important. The following section discusses key benchmarking techniques for assessing the security performance of LLMs in cybersecurity.

## 3. Benchmarks for Evaluating LLMs in Cybersecurity

To address these evaluation challenges, this section explores key benchmarking methods designed to assess the capabilities of LLMs in cybersecurity. In recent years, the rapid advancements in LLMs have opened new avenues for their application in cybersecurity. However, effectively evaluating the capabilities, limitations, and security implications of these models remains a critical challenge. To address this, researchers have developed a variety of benchmarking datasets specifically tailored for assessing LLM performance across diverse cybersecurity tasks. These benchmarks span a wide range of domains, including Industrial Control Systems (ICSs), network operations, debugging, code security, and adversarial vulnerability testing. Table 2 and Table 3 present a comparison of datasets used for evaluating LLMs in cybersecurity.

This section provides an overview of prominent evaluation benchmarks, each designed to measure specific aspects of LLM performance in cybersecurity. From assessing knowledge extraction and reasoning in ICS environments to testing the ability to detect and mitigate code vulnerabilities, these datasets represent the cutting edge of LLM evaluation in this domain. By leveraging these benchmarks, researchers and practitioners can gain valuable insights into the strengths and weaknesses of LLMs, guiding the development of more secure, efficient, and reliable AI-driven solutions for cybersecurity challenges. The following subsections detail the key features and objectives of each benchmark, highlighting their unique contributions to the field.

### 3.1. Cybersecurity in Industrial Control Systems

The SECURE [32] dataset is specifically crafted to evaluate the performance of Large Language Models (LLMs) in cybersecurity tasks within the domain of ICSs. It comprises six datasets aimed at assessing models’ abilities in knowledge extraction, understanding, and reasoning, leveraging industry-standard sources for realistic and practical scenarios. The benchmark provides a detailed analysis of seven advanced LLMs, highlighting their capabilities and limitations in addressing cybersecurity challenges. By focusing on ICS-specific tasks, SECURE delivers valuable insights into model reliability, fostering advancements in the development of LLMs as effective tools for cybersecurity applications.

### 3.2. Network Operations Evaluation

The NetEval [33] is a comprehensive evaluation dataset designed to assess the capabilities of pre-trained LLMs in Network Operations (NetOps). It includes 5732 multiple-choice questions across five distinct sub-domains, targeting both commonsense knowledge and inference abilities within the field. NetEval supports multi-lingual evaluation, enabling analysis of LLM performance in diverse linguistic contexts. The dataset has been used to evaluate 26 publicly available LLMs, revealing that GPT-4 achieves results comparable to human-level performance, while open models like LLaMA 2 demonstrate notable promise.

### 3.3. Debugging Capabilities of LLMs

The DebugBench [34] is a specialized benchmarking dataset designed to evaluate the debugging capabilities of LLMs. It consists of 4253 instances across C++, Java, and Python, covering four major bug categories and 18 minor types. The dataset was meticulously curated by collecting code snippets from the LeetCode community and introducing bugs using GPT-4, followed by rigorous quality checks to ensure reliability. DebugBench assesses LLM performance in zero-shot scenarios, revealing insights into debugging complexity, the impact of runtime feedback, and the relationship between debugging and code generation. This dataset aims to advance LLM development for debugging tasks.

### 3.4. Security Knowledge Assessment

The SecQA [35] dataset is a specialized dataset designed to evaluate the capabilities of LLMs in the field of computer security. It features multiple-choice questions derived from the “Computer Systems Security: Planning for Success” textbook, with content generated using GPT-4. The dataset is organized into two versions, v1 and v2, which progressively increase in complexity to test a wide range of skills and understanding. SecQA has been utilized to benchmark various LLMs, including GPT-3.5-Turbo, GPT-4, Llama-2, Vicuna, Mistral, and Zephyr, under both zero-shot and few-shot learning scenarios. This dataset offers a comprehensive evaluation framework for assessing how well these models grasp and apply security concepts, making it a valuable resource for advancing research in the domain of LLMs and cybersecurity.

The CyberMetric dataset [37] is a comprehensive benchmarking tool designed to evaluate the cybersecurity knowledge of Large Language Models (LLMs). It features four distinct subsets—CyberMetric-80, CyberMetric-500, CyberMetric-2000, and CyberMetric-10,000—comprising multiple-choice questions across key domains such as cryptography, reverse engineering, and risk assessment. Questions were generated using GPT-3.5 and Retrieval-Augmented Generation (RAG) techniques, drawing from authoritative sources like NIST standards, research papers, and publicly accessible books. Each question underwent rigorous validation by human experts, ensuring accuracy and relevance. The dataset has been used to benchmark 25 leading LLMs and was also tested with human participants for comparison. Results indicate that top-performing LLMs often surpass human performance in certain subsets.

### 3.5. Code-Generation Security

The SecurityEval [36] dataset is designed to assess the security of automated code-generation models, focusing on their ability to avoid generating vulnerable code. It consists of 130 samples that cover 75 distinct vulnerability types, each mapped to the Common Weakness Enumeration (CWE) framework. The dataset provides a practical benchmark for evaluating both open-source models like InCoder and closed-source models such as GitHub Copilot. By highlighting vulnerabilities in generated code, SecurityEval serves as a valuable tool for improving the reliability and safety of code produced by these models.

### 3.6. Foundational Knowledge in Cybersecurity

The SecEval [38] dataset is a pioneering benchmark designed to evaluate the cybersecurity knowledge of foundation models. It features over 2000 multiple-choice questions spanning nine critical domains, including software security, application security, cryptography, and network security. The dataset is developed using OpenAI’s GPT-4, leveraging information from credible sources such as textbooks, industry guidelines, and official documentation. Each question undergoes rigorous quality checks to ensure accuracy, diversity, and fairness, making SecEval a reliable resource for assessing and advancing AI capabilities in cybersecurity.

### 3.7. Python Code Security

The PythonSecurityEval [39] dataset is designed to advance research in code security by addressing vulnerabilities in real-world applications such as databases, websites, and operating systems. It introduces Feedback-Driven Security Patching (FDSP), an innovative approach where LLMs are guided by static code analysis to identify and fix security flaws in generated code. This dataset serves as a comprehensive resource for evaluating and improving the safety of code produced by LLMs, showcasing up to a 17.6% improvement in vulnerability mitigation compared to self-feedback methods. By covering diverse scenarios, PythonSecurityEval supports the development of safer and more reliable AI-driven code-generation solutions.

### 3.8. IT Operations Evaluation

The OpsEval [40] is a task-oriented benchmark designed to evaluate the capabilities of LLMs in the field of IT operations. It features a diverse set of 7184 multiple-choice questions and 1736 question-answering items, presented in both English and Chinese. The dataset addresses critical Ops tasks, including root cause analysis, operations and maintenance scripting, and alert summarization, catering to various ability levels. To ensure reliability, the questions were manually reviewed by domain experts. While 20% of the dataset is openly available to facilitate initial evaluations, the remaining 80% is kept private to prevent test leakage. Additionally, an online leaderboard allows real-time tracking and comparison of LLM performance, ensuring continuous benchmarking as new models emerge. This dataset provides a comprehensive foundation for assessing the effectiveness of LLMs in AIOps, while also exploring areas like model evaluation, QA accuracy, and hallucination mitigation.

### 3.9. Adversarial Code Vulnerabilities

The EvilInstructCoder [41] is a framework developed to evaluate the cybersecurity vulnerabilities of instruction-tuned Code LLMs against adversarial attacks. It features an Adversarial Code Injection Engine capable of generating and embedding malicious code snippets into benign datasets to simulate real-world threat scenarios. The dataset focuses on assessing the exploitability of state-of-the-art models like CodeLlama, DeepSeek-Coder, and StarCoder2 under diverse attack settings. Experimental results demonstrate that injecting a small percentage (0.5%) of poisoned data into the instruction-tuning datasets can lead to high attack success rates, highlighting critical security risks. This dataset underscores the urgent need for robust defensive strategies to safeguard AI coding assistants.

### 3.10. Cognitive-Level Cybersecurity Tasks

CS-Eval [42] is a publicly accessible benchmark specifically designed to evaluate the performance of LLMs in cybersecurity tasks. It encompasses 42 diverse categories, offering questions systematically organized into three cognitive levels: knowledge, ability, and application. The dataset is bilingual, supporting both English and Chinese, and draws its content from academic research trends and real-world industrial applications. CS-Eval provides valuable insights into the strengths and weaknesses of various LLMs, revealing that while GPT-4 excels overall, other models surpass it in specific subcategories. By analyzing performance trends over time, CS-Eval highlights the significant advancements in LLM capabilities for cybersecurity applications.

### 3.11. Coding Assistant Vulnerabilities

The CyberSecEval [43] is a benchmark designed to enhance the cybersecurity capabilities of LLMs when functioning as coding assistants. As one of the most comprehensive unified cybersecurity safety benchmarks available, it evaluates LLMs in two key areas: their likelihood to produce insecure code and their compliance when faced with requests to facilitate cyberattacks. Through the assessment of seven advanced models, including those from the Llama 2, Code Llama, and OpenAI GPT families, CyberSecEval reveals critical vulnerabilities and areas for improvement. The benchmark utilizes an automated pipeline for generating and evaluating test cases, offering a broad scope for analysis. Notably, the study highlights that more sophisticated models are more prone to generating insecure code, emphasizing the importance of integrating robust security mechanisms during their development. CyberSecEval provides actionable insights, equipping researchers and developers with tools to strengthen the safety and reliability of AI systems in cybersecurity contexts.

### 3.12. Code Security Evaluation

The LLMSecEval [44] dataset is designed to assess the security performance of LLMs in code-generation tasks. It contains 150 natural language prompts, each describing a code snippet vulnerable to one of MITRE’s Top 25 CWE. Accompanying each prompt is a secure implementation example, enabling comparative analysis of LLM-generated code. The dataset facilitates evaluation of how well LLMs can generate secure code from natural language descriptions, providing a practical framework for identifying vulnerabilities and encouraging secure coding practices.

### 3.13. Capture the Flag Challenges

NYU CTF Dataset [45] is a scalable, open-source benchmark designed to evaluate the performance of LLMs in solving cybersecurity Capture the Flag (CTF) challenges. Compiled from popular CTF competitions, the dataset includes diverse tasks and metadata tailored for LLM testing and adaptive learning. It supports advanced function calling and external tool integration, enabling a fully automated evaluation system with enhanced workflows. The dataset facilitates the assessment of five LLMs, encompassing black-box and open-source models, and compares their performance with human participants in interactive cybersecurity tasks. This benchmark provides a robust platform for advancing LLM capabilities in vulnerability detection, task automation, and real-world threat management.

The Dynamic Intelligence Assessment (DIA) [46] framework introduces an innovative approach to evaluating AI models by leveraging dynamic question templates and advanced metrics to address the limitations of static benchmarks. The accompanying dataset, DIA-Bench, spans various disciplines, including mathematics, cryptography, cybersecurity, and computer science, featuring diverse challenge formats such as text, PDFs, visual puzzles, and CTF-style tasks. By incorporating four novel metrics, DIA highlights gaps in model reliability and confidence, revealing frequent errors even with seemingly simple questions when presented in varied forms. Evaluations of 25 leading LLMs demonstrated challenges with complex tasks and unexpected inconsistencies in confidence levels, setting a new benchmark for assessing adaptive intelligence and self-awareness in AI systems.

### 3.14. Large-Scale Vulnerability Detection

The eyeballvul dataset [47] is a comprehensive benchmark designed to evaluate the ability of language models to detect vulnerabilities in large-scale codebases. It is sourced and updated weekly from publicly available open-source repositories, providing a dynamic and evolving testbed. The dataset includes over 24,000 documented vulnerabilities across 6000+ revisions and spans more than 5000 repositories. With a total size of 55 GB, it pairs each code revision with its corresponding list of known vulnerabilities, allowing precise evaluation of model performance. An LLM-based scoring system compares predicted vulnerabilities against the documented ones, ensuring a robust assessment of detection capabilities.

### 3.15. Cyber-Attack Attribution

The AttackER dataset [48] is the first dataset specifically designed to extract attribution information for cyber-attacks using Named Entity Recognition (NER) techniques. It aims to assist cybersecurity analysts in identifying attackers and implementing countermeasures by providing rich annotations that capture contextual details, including multi-sentence spans. This dataset addresses a critical gap in the domain by offering advanced tools to support attribution tasks, which are traditionally performed manually due to their complexity. Additionally, it demonstrates the potential of LLMs to enhance NER performance in cybersecurity, showcasing its utility in improving the accuracy and efficiency of cyber-attack attribution.

### 3.16. Expanded Cybersecurity Risks

CYBERSECEVAL 3 [49] is a comprehensive benchmark suite designed to evaluate the cybersecurity risks and capabilities of LLMs. It assesses eight distinct risks divided into two categories: risks to third parties and risks to developers and end-users. This iteration expands on prior benchmarks by incorporating offensive security capabilities, such as automated social engineering, scaling manual offensive operations, and autonomous offensive strategies. The dataset has been applied to Llama 3 and other cutting-edge LLMs, offering insights into their performance with and without mitigation measures, enabling a deeper understanding of their strengths and potential vulnerabilities.

Our results align with prior studies on AI-driven cybersecurity, particularly those analyzing LLM-based threat detection. For example, refs. [50,51] observed limitations in zero-shot LLMs for adversarial attack detection, a trend that we also confirmed. Additionally, ref. [52] identified dataset constraints in evaluating AI-driven cybersecurity tools, which our benchmarking process also highlighted. Compared to [53,54], our evaluation covers a broader range of security tasks, including adversarial robustness and multi-dataset validation, demonstrating the advantages of our methodology. While benchmarking AI models provides information on their cybersecurity capabilities, understanding the larger threat landscape is essential. The next section examines the most pressing cybersecurity issues that affect CNI.

## 4. Cybersecurity Issues

Cybersecurity issues significantly threaten critical infrastructure reliability, operation, and consistency. The interconnected nature of these systems and their reliance on digital technologies make them vulnerable to cyberattacks, which can have far-reaching effects on society.

**Figure 3:**
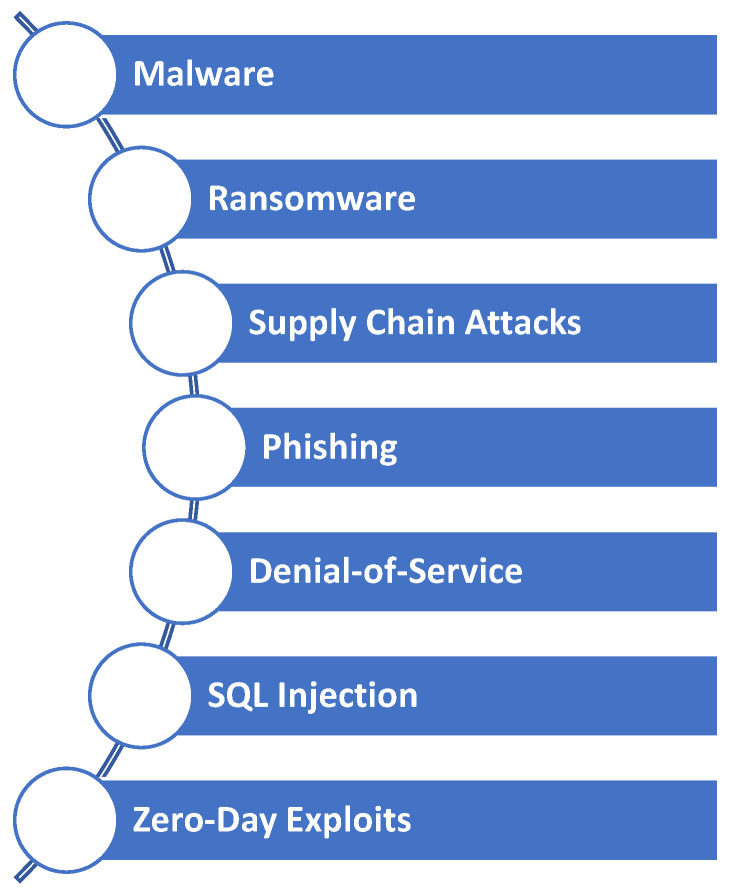
Some common and highly prevalent cyber threats towards critical national infrastructures.

One of the primary concerns regarding cybersecurity for critical infrastructures is the potential for malicious actors to infiltrate and disrupt essential systems. Cyber threats can range from simple phishing attempts to sophisticated malware injections and ransomware attacks [19], which all compromise the integrity and functionality of critical infrastructure networks. For example, a successful attack on an energy grid could result in widespread power outages, affecting millions of individuals and businesses. Figure 3 shows some common and highly prevalent cyber threats that CNIs should be aware of.

- *Malware:* Malicious software like viruses, worms, and Trojan horses can compromise the integrity, availability, and confidentiality of critical infrastructure systems. A malware program may be designed to steal sensitive data, disrupt operations, or allow attackers to take control of infrastructure assets remotely.
- *Ransomware:* Critical infrastructure has increasingly been targeted by ransomware attacks. The attacks can disrupt operations and demand large ransom payments, resulting in financial losses and outages.
- *Supply Chain Attacks:* A critical infrastructure often depends on third-party vendors for hardware, software, and services. The threat of supply chain attacks, where attackers compromise suppliers to gain access to target infrastructure, is becoming more common and difficult to detect.
- *Phishing:* Phishing attacks target employees or system users in an attempt to obtain sensitive information, such as login credentials or financial information. By impersonating legitimate entities, such as utility providers and government agencies, phishing emails or messages can gain access to critical infrastructure networks.
- *Denial-of-Service:* By overloading critical infrastructure systems with traffic, these attacks cause them to become slow or unresponsive. Multi-device DDoS attacks can disrupt essential services like communication networks and online utilities.
- *SQL Injection:* Databases are targeted by SQL injection attacks that exploit vulnerabilities in web applications. Attackers can manipulate SQL queries to access, modify, or delete sensitive data stored in critical infrastructure systems.
- *Zero-Day Exploits:* Zero-day exploits can take advantage of previously unknown vulnerabilities in software or hardware that have not yet been patched. These vulnerabilities are exploited by attackers to gain unauthorized access to critical infrastructure systems, steal data, or disrupt operations before security patches are available.

Moreover, a breach in one sector can cascade to others due to the interconnected nature of critical infrastructure. For instance, an attack on a transportation network could disrupt the supply chain, causing shortages of essential goods and services [55]. The interconnectedness of our world amplifies cybersecurity issues and highlights the need for comprehensive protection measures.

As IoT devices become integral to critical infrastructure, they introduce new vulnerabilities due to their large-scale deployment and often limited security mechanisms. IoT-specific threats include firmware exploits, device hijacking, and distributed denial-of-service attacks leveraging botnets [16]. These devices frequently lack strong authentication, making them attractive targets for adversaries aiming to disrupt operations or gain unauthorized access.

Additionally, the rise of AI-driven attacks presents novel challenges, such as AI poisoning and model-inversion attacks. AI poisoning occurs when adversaries inject manipulated data into training datasets to alter the behaviour of machine learning models, potentially leading to the misclassification of security threats. On the other hand, model-inversion attacks allow adversaries to reconstruct private training data from exposed AI models, posing risks to sensitive critical infrastructure information.

Cybersecurity issues can also undermine the reliability and consistency of critical infrastructure operations. A security breach erodes trust in these systems, which are crucial for effective functioning. This may cause stakeholders to hesitate to rely on critical infrastructures, leading to disruptions in service delivery and economic instability. In addition, recovering from cyber attacks can be costly, further straining resources and disrupting operations. According to the European Union Agency for Cybersecurity (ENISA), protecting critical infrastructure requires a multi-layered cybersecurity strategy that involves risk assessment, incident response planning, and regulatory compliance. ENISA emphasizes the importance of cybersecurity resilience in critical sectors such as energy, healthcare, and finance, where disruptions can have cascading effects on public safety and economic stability. Their guidelines recommend enhanced cooperation between governments and private stakeholders to address evolving threats [56].

In general, it is important to mitigate the risk of these attacks by prioritizing cybersecurity and implementing comprehensive protection measures. Failure to address these challenges effectively could severely affect national security, economic stability, and social well-being. Mitigating these emerging threats requires implementing secure model training frameworks, robust authentication for IoT devices, and continuous monitoring with anomaly-detection systems tailored to AI and IoT environments. Addressing cybersecurity issues requires a strong foundation in trust, privacy, and resilience. The next section explores these core principles and how they contribute to the security of CNI systems.

## 5. Trust, Privacy, and Resilience

CNIs represent vital systems essential for the functioning of a nation or region, imposing the need to adhere to stringent privacy standards. The specific privacy, trust and resilience, in most cases, requirements applicable to CNIs vary based on location and characteristics and are subject to diverse regional standards, laws, and regulations. Examples include the General Data-Protection Regulation (GDPR) in the European Union [57], the Health Insurance Portability and Accountability Act (HIPAA) in the United States [58], and the Personal Data-Protection Act (PDPA) in Singapore [59].

To protect CNIs, privacy requirements aim to ensure the integrity, confidentiality, and availability of associated information and systems. Key considerations include confidentiality and data protection, restricting system access to authorized personnel, compliance with privacy laws like GDPR and HIPAA, and implementing robust network security measures like cryptography and AI. Access to CNIs is secured through physical security measures like video surveillance and alarms. The resilience of CNI systems is assured through comprehensive recovery and data backup processes, employing techniques like data replication and cloud backup. Emerging technologies such as blockchain and AI are increasingly integrated into CNI systems to enhance security and resilience. Blockchain offers a decentralized and immutable ledger that ensures the integrity and transparency of data transactions, making it particularly suitable for identity management, secure data sharing, and tamper-proof documentation of critical operations [60,61]. For example, smart contracts on blockchain platforms can automate and enforce security policies, ensuring compliance with regulatory standards and reducing the risk of human error. AI is utilized to predict and identify potential cyber threats in real time. By analyzing vast amounts of data from network traffic, system logs, and threat intelligence feeds, AI systems can detect anomalies and respond to threats faster than traditional methods [62]. Additionally, AI-driven automation in incident response and recovery processes enhances the efficiency and effectiveness of maintaining CNI operations during and after cyber incidents [63]. These technologies increase CNI systems’ security posture and improve their resilience against evolving cyber threats.

To strengthen cybersecurity, CNIs deploy a spectrum of techniques encompassing network security, application security, data security, identity and access management, and risk management. These measures, including advanced encryption standards and innovative solutions proposed in the literature [64], collectively contribute to a multi-layered defence against cyber threats. Periodic monitoring, audits, and a continuous commitment to privacy compliance are integral components to guarantee the efficacy of these security measures, fostering the overall resilience and reliability of CNIs.

To build trust and resilience, CNI security must balance protection against cyber threats with system safety. The following section examines the interplay between safety and security, presenting an integrated approach to CNI protection.

## 6. Securability

Scholars who conduct research in security or safety tend to address each field independently of the other. We strongly believe that these fields are interdependent, and based on some recent works, we present the current research in this area. The co-analysis of safety and security can be classified into two major categories: integrated strategy and unified strategy [65]. The main difference between integrated strategy and unified strategy lies in the approach they take when they combine security and safety, the former focusing on integrating the results while the latter on the co-analysis of the system. As stated in [66], safety and security co-analysis (SSCA) could benefit accident prevention in the transportation sector. To differentiate between security attacks and safety problems, scholars classify the events that lead to threats or hazards. They also state that security risks come from deliberate actions while safety risks come from mistakes or errors [67], neglecting that mistakes of the users initiate many security attacks.

The idea of including a probabilistic model of the behaviour of a part or the whole system in the form of suspected failures or faults could provide a better picture of the system in the analysis and a prediction of future states. For example, let us imagine that we are trying to analyze the behaviour of a system from a high-level perspective when the system also has a disaster recovery facility. For disaster recovery to work, the data and computer processing must be replicated at an off-site location that is unaffected by the incident. An organization needs to recover lost data from a backup location if the servers go down due to a natural disaster, equipment malfunction or cyber attack. To maintain operations, a business should also be able to move its computer processing to this remote location so that it can continue to provide its services to its customers.

The main system is represented as MS and the disaster recovery site as DR. If we work on an abstract level, we can represent the states of the system using a Markov Chain, where:

- State $S_{o}$ is when both the system and the DR site are operating normally;
- State $S_{1}$ is when the system is down due to a malfunction or attack;
- State $S_{2}$ is when the DR site is switched off;
- State $S_{3}$ is when both S and DR are switched off.

Here, $\lambda_{MS}$ is the failure rate of the system, and $\lambda_{DR}$ is the failure rate of the disaster recovery site. The transition from state $S_{0}$ or state $S_{1}$ to state $S_{2}$ occurs at rates $\mu_{MS}$ and $\mu_{DR}$ respectively, which represent the repair/recovery rate of the system/DR.

**Figure 4:**
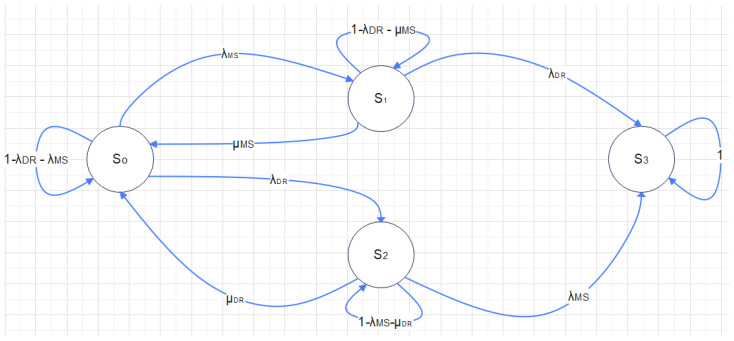
A Markov Chain of an MS/DR system.

For an organization to offer services around the clock, the failure rate (λ) must be lower than the recovery rate (μ). Using the Markov model from Figure 4, we can calculate the MTTF or MTTA (Mean Time to Attack) and the MTTR depending on the model used. The correct values for these rates require a thorough analysis of the system components and their interdependencies and an up-to-date assessment of the threats. This analysis is demanding and must be performed using a top-down approach in several steps. The main system can be divided into subsystems. A state transition diagram for each subsystem must be created, together with a general model that represents the dependencies between the subsystems in the general form r out of n (r out of n:G). In this model, at least the r subsystems or elements must be in a good state for the system to be operational. When incorporating cybersecurity into this reliability analysis, the calculation of the failure probability of each component must include failures as well as possible attacks.

### Collaborative Intelligence for Privacy-Preserving CIP

Ensuring data privacy in CIP is a major challenge due to the sensitive nature of operational data. Traditional AI models require centralized data collection, increasing security risks. Collaborative intelligence techniques provide a solution by enabling AI models to learn from decentralized datasets while maintaining privacy.

Federated learning is a decentralized learning approach in which AI models are trained across multiple devices or institutions without exchanging raw data. This technique is widely used in IoT security and industrial control systems to prevent exposure to sensitive data while improving cybersecurity capabilities [51,52]. In CIP, federated learning allows different network operators to collaborate in detecting cyber threats without sharing raw traffic data.

Secure Multi-Party Computation (SMPC) is another crucial technique for ensuring privacy when processing sensitive data in CIP applications [68]. Unlike federated learning, which distributes model training across multiple devices, SMPC enables multiple parties to collaboratively compute a function over their inputs while keeping the inputs private. It ensures that data can be processed securely without being exposed, even in multi-stakeholder environments—such as smart grids, industrial control systems, and transportation networks. Homomorphic encryption can further enhance the feasibility of SMPC in real-time CIP operations. Future research can explore hybrid models integrating federated learning and SMPC to achieve stronger privacy guarantees while maintaining model performance.

Transfer learning helps AI models adapt to new environments by leveraging pre-trained knowledge [69]. This technique is particularly useful for healthcare security and smart grids, where real-world datasets are often limited due to privacy concerns [70]. By using transfer learning, models trained on general cybersecurity data can be fine-tuned for specific CIP domains without requiring extensive data sharing.

Multi-agent learning involves multiple AI agents working together to enhance distributed system security. This method is beneficial for anomaly detection and cyber-attack defence, where multiple agents can monitor network activity and respond to threats in real time [51,71]. Multi-agent learning enables intelligent coordination among different infrastructure components to improve resilience against cyberattacks. These collaborative learning techniques provide strong privacy-preserving capabilities for AI applications in CIP. Future research can explore hybrid approaches integrating federated learning, transfer learning, and multi-agent learning to enhance cybersecurity while ensuring compliance with data-protection regulations.

With a clearer understanding of securability challenges, the role of emerging AI technologies is becoming more important in addressing these issues. The next section explores how Generative AI and LLMs can enhance the security and resilience of CNI.

## 7. Generative AI and Large Language Models for Critical Infrastructure Protection

The deployment of Generative AI and LLMs in CIP is more than theoretical; it is a burgeoning reality with real-world applications that demonstrate the potential of these technologies. This section highlights specific examples of how LLMs have been utilized to enhance the resilience and security of critical infrastructure, from energy grids to water-treatment facilities [72,73].

### 7.1. LLM Lifecycle for Critical Infrastructure Protection

**Figure 5:**
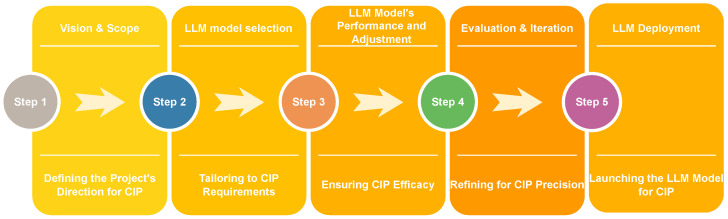
The steps of Generative AI and LLM lifecycle for Critical Infrastructure Protection.

For an application focused on CIP using LLMs, tailoring the lifecycle to emphasize CIP’s unique challenges and requirements—such as security, resilience, and domain specificity—is critical [74]. In this sub-section, we discuss the LLM lifecycle for CIP, which is based on the following five steps, as presented in Figure 5.

#### 7.1.1. Vision and Scope: Defining the Project’s Direction for CIP

- *Objective Clarification:* We establish the model’s role in protecting critical infrastructure. ‘Will it analyze threat intelligence, aid vulnerability assessments, or assist in emergency response?’ Setting a clear, CIP-focused objective will guide the development process.
- *Scope Determination:* We identify which critical infrastructure sectors the LLM will focus on, such as energy, water, and transportation. Different sectors may require different types of data and domain knowledge.

#### 7.1.2. Model Selection: Tailoring to CIP Requirements

- *Security and Reliability:* We choose or develop a new model emphasizing security and data privacy, which are essential for CIP applications.
- *Domain Adaptation:* We decide whether to adapt an existing LLM or train a new one with a dataset enriched with CIP-related content.

#### 7.1.3. Model’s Performance and Adjustment: Ensuring CIP Efficacy

- *Performance Assessment:* We evaluate the model’s ability to identify, classify, and predict threats to critical infrastructure.
- *Adjustment for CIP:* Focus adjustments on enhancing the model’s capability to deal with the specific nuances of critical infrastructure threats. Hence, this could involve prompt engineering with CIP-specific prompts or further fine-tuning on targeted datasets.

#### 7.1.4. Evaluation and Iteration: Refining for CIP Precision

- *CIP-Specific Metrics:* We use evaluation metrics that reflect the model’s performance in a CIP context—threat-detection accuracy, response speed, and ability to work with domain-specific data.

Threat-detection accuracy is a key metric for evaluating the effectiveness of AI-driven security mechanisms in CIP. It measures the system’s ability to correctly identify and classify threats while minimizing false positives and false negatives. It is formally defined as:

(12) $$\mathrm{Threat}-\mathrm{Detection}\;\mathrm{Accuracy}=\frac{TP}{TP+FN}$$

where TP, true positives, represents the correctly identified threats, and FN, false negatives, represents missed threats. This metric is critical in assessing how well an AI-based system can differentiate between normal activities and malicious threats, reducing the likelihood of undetected attacks.

Response speed quantifies how quickly an AI-driven security system reacts to detected threats. It is typically measured as the time elapsed between threat detection and the execution of a mitigation action. This metric can be represented as:

(13) $$\mathrm{Response}\;\mathrm{Speed}=T_{mitigation}-T_{detection}$$

where $T_{mitigation}$ is the timestamp when mitigation actions are initiated, and $T_{detection}$ is the timestamp when the threat was first identified. Faster response speeds indicate a more effective incident response system capable of minimizing potential damage to critical infrastructure.

#### 7.1.5. LLM Deployment: Launching the LLM Model for CIP

Once deployed, we must establish mechanisms for ongoing monitoring of the model’s effectiveness and updates to maintain its relevancy against evolving threats to critical infrastructure.

### 7.2. Predictive Analysis and Threat Intelligence: The Case of Energy Grid-Protection

LLMs can be leveraged in the energy sector to detect potential cyber-attacks on power grids. For example, a company might utilize models like GPT-4 (Generative Pre-trained Transformer-4) to analyze and interpret extensive unstructured text data from online forums, threat reports, and system logs to predict and identify potential cyber threats, including phishing attacks or malware aimed at energy grid systems [75]. However, processing company data within an external data centre, such as OpenAI’s, raises privacy concerns. Developing a sector-specific LLM model and deploying it within the company’s data centre can enhance the protection of sensitive data.

### 7.3. Automated Incident Response: Enhancing Pipeline Security

BERT [76], known for its deep understanding of language context, is particularly useful for parsing and extracting specific information from incident reports, security logs, or communication between stakeholders involved in critical infrastructure. For example, a transportation authority could use BERT to quickly sift through incident reports following a security breach in a public transportation network, identifying common patterns or vulnerabilities that need immediate attention. Thus, it would speed up the response time and ensure the safety and reliability of transportation services.

### 7.4. Enhancing Communication and Coordination: Water-Treatment Facility Case Study

T5 [77] can convert language-based tasks into a unified text-to-text format, making it exceptionally suitable for generating compliance and policy documentation vital for critical infrastructure sectors. A water-treatment facility might leverage T5 to automate the creation of compliance reports based on new regulatory guidelines and operational data. This ensures accuracy and adherence to legal requirements and significantly reduces the administrative burden, allowing staff to focus on operational excellence and system integrity.

### 7.5. Challenges and Considerations

In the context of applying Generative AI and LLM models for critical infrastructure, integrating these models poses several open challenges. Each challenge requires careful consideration and innovative solutions to ensure the effective and secure application of LLMs. Below, we explore these challenges in more detail.

#### 7.5.1. Building an Instruction Cybersecurity Dataset

One of the primary challenges in leveraging LLMs for critical infrastructure is developing a comprehensive and relevant cybersecurity dataset. Critical infrastructure systems are highly complex and often proprietary, making it difficult to gather real-world data for training purposes. Additionally, the dataset must be diverse enough to cover various cyber threats and attack vectors unique to critical infrastructure sectors. Ensuring the dataset’s quality, relevance, and privacy compliance also poses significant challenges, as it must be constantly updated to reflect evolving cyber threats. The structure of the Alpaca dataset can be adapted for building an instruction cybersecurity dataset [78] as shown in Table 4.

#### 7.5.2. Pre-Training Models

Pre-training LLMs for critical infrastructure applications also involves several challenges, including selecting appropriate pre-training tasks that align with cybersecurity contexts. The sheer volume of data required for effective pre-training and the computational resources needed are substantial. Moreover, the model must be trained to generalize across critical infrastructure sectors without compromising sector-specific requirements.

Developing pre-trained LLMs for CIP involves a meticulous process that starts with collecting and preparing high-quality, domain-specific datasets. In the context of critical infrastructure, this includes gathering extensive data from cybersecurity reports, threat intelligence feeds, and technical documents related to infrastructure systems. The preprocessing and cleaning of this dataset are vital steps, ensuring that irrelevant, duplicate, or sensitive information is removed, thereby refining the dataset to contain only the most relevant and high-quality data for training purposes [79,80].

Following the preparation of a meticulously curated dataset, the model architecture must be defined, considering critical infrastructure security’s specific needs and challenges. This includes selecting the appropriate LLM architecture—such as variants of GPT or other autoregressive models—and adjusting hyperparameters to optimize performance for the unique context of critical infrastructure. The training involves teaching the LLM to predict the next word in a sequence and enabling it to understand complex cybersecurity concepts and the nuances of different threat vectors affecting critical infrastructure [81,82].

#### 7.5.3. Supervised Fine-Tuning

**Figure 6:**
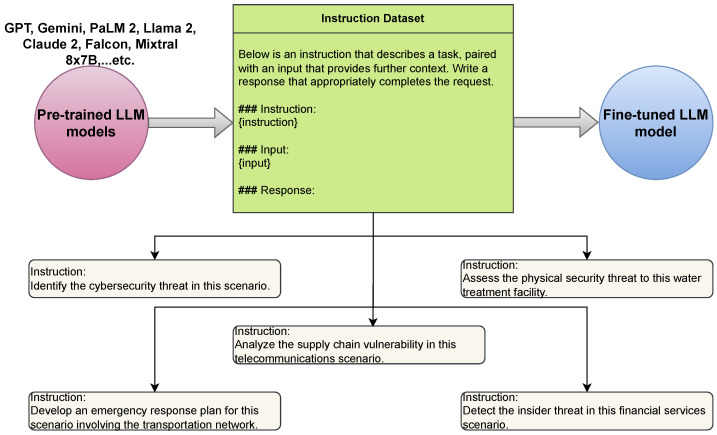
Instruction fine-tuning LLM models for Critical Infrastructure Protection.

Supervised fine-tuning for LLMs in CIP involves updating pre-trained language models with specific, labelled datasets to perform targeted tasks more efficiently [83], as presented in Figure 6. This process, distinct from unsupervised methods, enhances models’ ability to interpret and react to nuanced requirements within the critical infrastructure domain. By employing labelled examples tailored to the unique challenges of infrastructure security, such as threat detection or system diagnostics, LLMs can offer more precise and relevant responses, improving overall security measures [84]. Therefore, the limitation of supervised fine-tuning in applying LLMs for CIP lies in its reliance on high-quality, labelled datasets. This requirement can pose challenges in scenarios where such data are sensitive or expensive, potentially limiting the model’s learning capability and adaptability to new or evolving threats within critical infrastructure sectors [85].

Exploring fine-tuning methodologies beyond the conventional supervised approach can offer nuanced benefits and challenges. Transfer learning and task-specific fine-tuning stand out for their potential to adapt LLMs like GPT and BERT to specialized tasks, leveraging pre-existing vast datasets for efficiency and accuracy in targeted applications. However, these methods can also introduce risks such as catastrophic forgetting, where a model’s performance on non-fine-tuned tasks deteriorates [86].

Multi-task learning and sequential fine-tuning present solutions to broaden an LLM’s capabilities across multiple tasks or gradually specialize its knowledge, mitigating the drawbacks of single-task focus [87]. While demanding extensive datasets, these approaches enable the creation of versatile models capable of handling diverse tasks relevant to safeguarding critical infrastructure, thus offering a balanced strategy to exploit LLMs’ strengths while addressing their limitations [88].

#### 7.5.4. Reinforcement Learning from Human Feedback

Reinforcement Learning from Human Feedback (RLHF) [89] involves enhancing Generative AI and LLM by incorporating direct human feedback into the learning process. This method has significantly improved LLMs’ relevance, accuracy, and ethical considerations, particularly in applications like chatbots. Integrating RLHF into LLM training allows models to understand better by aligning model outputs more closely with human preferences and expectations. This approach is especially beneficial for CIP, where nuanced understanding and accurate, reliable communication are paramount. Adopting RLHF can enable more effective monitoring, threat detection, and incident response, thereby enhancing the resilience and security of critical infrastructure systems. Therefore, this adoption faces challenges such as defining appropriate reward functions that accurately reflect critical infrastructure systems’ priorities and safety requirements.

#### 7.5.5. Quantization

Quantization offers a pathway to reducing the computational demands of deploying LLMs in critical infrastructure settings. Several techniques exist for quantizing LLMs to 4-bit precision. Examples include QuaRot [90], GPTQ [91], AWQ [92], SqueezeLLM [93], AQLM [94], and llama.cpp with GGUF, all of which are well-regarded methods compatible with numerous frameworks. However, challenges arise in maintaining model accuracy and performance under reduced precision. Ensuring that the quantized models can reliably detect and respond to cyber threats without false positives or negatives is paramount. Balancing model size and computational efficiency with the need for real-time, high-performance decision-making in critical infrastructure contexts is a significant challenge.

Beyond quantization, additional lightweight ML inference techniques can further enhance efficiency and reduce computational overhead. For instance, early-exit strategies allow models to stop processing earlier when confidence in a prediction is high, minimizing latency while maintaining reliable outputs [95]. Network pruning reduces the complexity of neural networks by eliminating less significant connections, leading to smaller models with faster inference times, making them ideal for constrained environments. Knowledge distillation transfers knowledge from larger, more complex models to smaller ones, enabling them to retain similar accuracy while significantly decreasing resource demands. These complementary approaches, alongside quantization, contribute to improving model efficiency and adaptability in CIP, ensuring robust performance even in environments with limited computational resources.

#### 7.5.6. Retrieval-Augmented Generation

Integrating RAG technology into the CIP domain introduces a transformative approach to safeguarding vital assets such as power grids, water systems, and communication networks [96]. Unlike traditional language models that excel in general tasks but lack the depth for specialized applications, RAG’s architecture—which combines an information retrieval component with a text-generation model—perfectly aligns with critical infrastructure security’s complex and dynamic nature. By enabling real-time access to external databases and the latest research on threats and vulnerabilities, RAG ensures the production of contextually relevant and factually accurate responses grounded in the most current information available. This capability is crucial for CIP, where rapidly assimilating and acting upon up-to-date intelligence can mean the difference between the regular operation of essential services and a potentially catastrophic failure.

The methodology introduced by Meta AI researchers [97] can involve fine-tuning a pre-trained model with a comprehensive index of documents relevant to critical infrastructure, offers a tailored solution for enhancing threat intelligence, vulnerability assessments, and incident response strategies. For example, RAG can be leveraged to analyze threat actor tactics, techniques, and procedures, assess the impact of potential vulnerabilities on critical systems, and generate informed recommendations for mitigating risks. The model’s strength in producing factual, specific, and diverse outputs significantly improves verifying facts and combating misinformation related to threats against critical infrastructure.

#### 7.5.7. Inference Optimization

The optimization of Generative AI and LLMs for inference in critical infrastructure is challenging. The techniques involve managing such models’ extensive compute and memory requirements, including optimizing the attention mechanism and managing memory more effectively through batching [98,99,100], key-value caching [101], and model parallelism [102,103]. These optimizations are crucial for deploying LLMs in real-world applications, including critical infrastructure, where efficient, reliable, and fast processing of large volumes of data is essential. To apply these concepts to critical infrastructure, we need to focus on customizing model parallelism and memory-management techniques to suit critical systems’ specific needs and constraints, ensuring that LLMs can be used effectively without compromising the performance or security of these vital services.

Ensuring the scalability of AI-driven CIP systems is critical due to the large data volumes and real-time processing constraints in critical environments. Key techniques include model parallelism, where deep learning models are distributed across multiple GPUs or TPUs to balance computational loads, and batching methods to optimize memory use and reduce latency. Additionally, techniques such as key-value caching and retrieval-augmented generation enable more efficient inference by dynamically retrieving relevant information from external sources, minimizing computational overhead. These optimizations enhance the deployment feasibility of AI models for large-scale CIP applications while ensuring real-time responsiveness and cost-efficiency.

While Generative AI provides significant advantages in threat analysis and response, Agentic AI introduces more autonomous decision-making capabilities. The next section discusses how Agentic AI can proactively mitigate operational risks in CNI.

## 8. Agentic AI for Critical Infrastructure Protection

Agentic AI describes a sophisticated AI system capable of autonomous action, real-time adaptation, and multi-step problem-solving aligned with specific contexts and objectives. Agentic AI offers a transformative framework for CIP in an era of sophisticated cyber-physical threats and evolving operational complexities [104]. Specifically, Agentic AI enables proactive defence and resilience in real time by autonomously learning, adapting, and orchestrating multi-step mitigation strategies with minimal human oversight. Table 5 compares Traditional CIP and Agentic AI-enabled CIP, underscoring the key enhancements of agentic architectures for safeguarding critical infrastructure.

### 8.1. Real-Time Anomaly Detection and Threat Mitigation

Unlike static rule-based solutions, Agentic AI agents utilize reinforcement learning to adapt detection thresholds dynamically [105]. The process continuous data streams—from sensor arrays, industrial control systems, or cybersecurity logs—and isolates legitimate anomalies in near real time. Agentic AI will integrate data from disparate sources, such as operational technology (OT) sensors, IT networks, and external threat intelligence feeds, to create a unified, contextualized view of potential incidents [106,107].

### 8.2. Intelligent Incident Response and Recovery

AI agents immediately identify, plan, and execute the necessary remediation steps upon detecting an incident (e.g., a ransomware attack on a power grid controller). They can isolate compromised segments, enforce automated fail-safes, or initiate patching protocols without waiting for manual commands [108]. Dynamic workflow agents autonomously orchestrate tasks such as rerouting power distribution, updating network configurations, and conducting rapid root-cause analysis. Human operators are kept in the loop for oversight, but the system can run end-to-end when seconds count [109].

### 8.3. Proactive Resilience and Predictive Maintenance

Sensor data and operational logs are fed directly into machine learning models, enabling early detection of wear and tear or performance anomalies. With foresight into potential mechanical failures, agents schedule preventive maintenance during off-peak hours and maintain a high availability of critical assets [110]. By observing outcomes (e.g., how quickly a system recovers after specific remediation steps), AI agents improve over time, refining the accuracy of predictions and the effectiveness of incident response strategies [111].

### 8.4. ML-Assisted Protection in Critical Infrastructure

The use of ML in CIP has increased significantly due to its ability to detect cyber threats, prevent system failures, and enhance operational security. ML-powered security models are applied across various industries, including industrial control systems, mission-critical services, transportation, and cyber defence. ML-based intrusion-detection systems play a key role in monitoring and protecting industrial control networks. These systems help detect anomalous activities, cyber threats, and system failures in real time [112]. For example, ref. [53] explores various ML-based detection models for industrial control systems security. AI-driven security models enhance mission-critical service management by automating threat detection, optimizing network performance, and preventing disruptions. Ref. [113] presents an ML-based security framework for smart city infrastructure and emergency response systems. Moreover, ML models are increasingly used for predictive maintenance, cybersecurity, and safety monitoring in aviation, railways, and autonomous vehicles. AI-based security frameworks help detect anomalies and prevent cyber threats in transportation networks, as discussed in [114]. ML-based models contribute to automated attack detection, multi-layered defence, and adaptive security in CIP. Ref. [115] highlights defensive machine learning techniques that enhance proactive threat mitigation and cyber resilience. These examples demonstrate how ML-powered solutions enhance security, resilience, and reliability in critical infrastructure. Future AI-driven cybersecurity advancements will improve real-time threat response and adaptive defence mechanisms in CIP.

### 8.5. Automated Policy Enforcement and Compliance

Agentic AI incorporates domain-specific knowledge, such as NERC CIP standards for power systems or ISO 27001 for information security, to ensure that actions stay within compliance thresholds automatically [116]. Agents can generate evidence for compliance audits on demand and archive all actions, decision rationales, and data flows. This continuous compliance monitoring aids in both everyday governance and in-depth forensic investigations [117].

### 8.6. Multi-Stakeholder Collaboration and Incident Coordination

Suppose a power grid malfunction threatens a region. In that case, specialized AI agents can instantly coordinate across multiple teams: public utilities, government agencies, and private contractors, ensuring everyone has the latest threat intelligence and situational updates [118]. The modular nature of Agentic AI allows integration with industry-specific monitoring systems, environmental controls, and other legacy platforms. It effectively functions as a unified intelligence layer, eliminating data silos and promoting joint situational awareness [119].

### 8.7. Ethical, Secure, and Trustworthy AI for CIP

While decisions may be executed autonomously, logs and decision outlines are accessible to human operators for auditing or regulatory needs. This transparency fosters trust in AI-driven CIP processes. Agentic AI itself must be secured. Methods such as AI agent identity management, cryptographic data protection, and rigorous endpoint security help mitigate the risk of AI being hijacked or manipulated by adversaries [120].

Regulatory compliance is critical in ensuring the ethical deployment of AI systems in CIP. Frameworks such as the GDPR enforce strict data protection and privacy measures, which impact how AI-driven security systems handle sensitive infrastructure data. Similarly, the Network and Information Systems (NIS) Directive orders enhanced cybersecurity measures for operators of essential services, aligning closely with AI-enabled CIP strategies. Beyond Europe, initiatives such as the US Cybersecurity Executive Order and international ISO/IEC 27001 standards establish global best practices for AI security in critical infrastructure. Addressing ethical concerns also involves defining clear accountability for AI-driven decisions, particularly in automated response systems where liability in case of failure remains an open issue. Future advancements in explainable AI and standardized auditing mechanisms will be necessary to ensure compliance, transparency, and fairness in AI-powered CIP frameworks.

### 8.8. AI Ethics

Several studies in the literature examine the vulnerabilities and advanced manipulation tactics of GenAI. Investigating these vulnerabilities underscores the major security risks associated with using advanced AI technologies, such as the potential for bypassing security measures through the RabbitHole attack and compromising data privacy through rapid injection [121].

However, the use of personal data by AI not only raises privacy concerns but can also undermine transparency for users of online services. This lack of transparency is intensified by the fact that algorithms can be so complex that they are often described as a “black box”. While there is broad consensus on the need for ethical AI, there is less agreement on what ethical AI should look like in practical terms [122].

As stated in [123], the emerging ethical challenges posed by generative Artificial Intelligence (AI) technologies stress the need for interdisciplinary collaboration and the creation of strong ethical frameworks. Concerns such as deepfakes, misinformation, biases, privacy, and the amplification of societal inequalities illustrate the complex relationship between technological progress and ethical responsibilities.

Finally, GenAI has the potential to drastically reshape offensive cyber tactics. Microsoft and OpenAI have reported early examples of AI being exploited by state-backed threat actors [124]. Research in [125] demonstrates that ChatGPT could be used to generate social engineering attacks, phishing schemes, automated hacking, attack payloads, malware, and polymorphic malware. While AI-powered tools such as PentestGPT are designed for legitimate, productive purposes, there is also the risk that malicious actors could replicate similar models to automate unethical hacking activities.

### 8.9. Bias Mitigation in AI for CIP

AI models used in CIP can show biases due to imbalanced training datasets. This issue can lead to inequitable outcomes, where certain infrastructure components, geographic locations, or user groups receive disproportionate attention or resources. Bias in AI models is particularly concerning in CIP scenarios, as it may result in misclassification of threats, inadequate response measures, or unfair allocation of security resources. Several techniques can mitigate AI bias in CIP applications:

- Data rebalancing ensures that diverse and representative datasets for training AI models, using techniques such as synthetic data augmentation and resampling methods to balance underrepresented cases.
- Fairness-aware model training includes bias correction algorithms, such as adversarial debiasing and reweighting methods, to ensure that the AI system does not systematically favour or neglect specific categories.
- Regular auditing of AI models for bias using explainable AI techniques identify and correct potential discriminatory patterns.
- Active learning continuously updates AI models with real-world data to improve their adaptability and reduce bias over time.

These techniques assist in ensuring that AI-driven CIP models provide consistent and equitable security assessments, reducing systemic biases and increasing trust in AI-based decision-making.

Authors in [126] argue that machine learning models are inherently designed to discriminate and that access to personal attributes actually helps reduce bias. They claim that models require knowledge of protected characteristics to function effectively, suggesting that legal restrictions may unintentionally foster bias rather than eliminate it. Unconscious can significantly affect individuals and groups, sometimes even reinforcing existing biases. In 2018, Amazon tested an AI recruitment tool designed to use machine learning to search the web for potential candidates, rating them on a scale from 1 to 5 stars. The tool was trained using a dataset of CVs submitted over a 10-year period aimed at identifying top candidates. However, Amazon discontinued the tool after discovering that it systematically downgraded women’s CVs for technical roles like engineering and software development.

With the advancement of technology, AI integration in CNI protection will continue to evolve. The final section highlights future directions and emerging trends shaping the next generation of cybersecurity solutions for critical infrastructure.

## 9. Future Directions

As CNIs evolve, adopting cutting-edge technologies becomes crucial to handle emerging threats and challenges. Table 6 presents key future directions in CIP and summarizes their overarching impact on resilience, security, and operational efficiency. Each direction highlights a novel technology or strategy—from digital twins to Agentic AI—that can bolster critical assets against escalating threats. By adopting these approaches, stakeholders can proactively address challenges posed by emerging cyber-physical risks, ensuring the continuous provision of essential services. In this section, we explore future directions that promise to enhance the resilience and security of critical infrastructures [127].

One innovative approach to CNI protection is the use of digital twins. It offers a paradigm shift in critical infrastructure systems protection, management, and monitoring [131,132]. Proactive mitigation measures can be implemented by simulating and evaluating the consequences of cyberattacks or system failures before their occurrence, thanks to the capability of digital twins. Virtual replicas of real assets and systems provide CNI stakeholders with previously unattainable insight into their operations, weaknesses, and possible sites of failure. Furthermore, by utilizing the sophisticated analytics and simulation capabilities of the digital twin-empower critical infrastructure, CNI operators can anticipate and reduce risks, improve performance, and simplify maintenance tasks. Integrating digital twin technology into CIP strategies promises to revolutionize the real-time monitoring, managing, and safeguarding of critical assets.

With the ability to perform complex calculations at speeds exponentially faster than classical computers, quantum computing promises to revolutionise threat detection, vulnerability assessments, and encryption methodologies. Quantum computing can enhance threat detection, risk analysis, and system optimisation for CIP. Critical infrastructure operators can maximise efficiency while minimising risks thanks to quantum algorithms’ ability to solve optimisation issues like resource allocation and network routing. Additionally, massive databases can contain hidden patterns that quantum machine learning algorithms can find, providing proactive threat intelligence and flexible security solutions.

With the rise of quantum computing, traditional encryption methods can be easily decrypted by quantum algorithms. Therefore, developing quantum-resistant encryption algorithms and cryptographic protocols is crucial to guarantee the long-term security of CNIs. Quantum encryption techniques provide secure communications and data transmission channels against even the most sophisticated cyber attacks [133]. Quantum encryption and cryptography methods have enormous potential to provide ultra-secure communication networks, protect critical infrastructures from malicious actors, and secure the confidentiality and integrity of sensitive information throughout CNIs. While quantum computing offers promising capabilities in threat detection, risk analysis, and encryption, its practical deployment in CIP remains limited due to hardware constraints and the need for stable quantum processors. Currently, most quantum applications are experimental and conducted in controlled environments. Widespread adoption of quantum security techniques, such as quantum-resistant encryption, will likely require advancements in error correction, fault-tolerant quantum computing, and improved quantum hardware scalability. It is expected that significant real-world applications in CIP may emerge within the next decade as quantum technology matures.

Augmented reality (AR) technologies hold the potential to revolutionise operator interaction and visualisation of critical infrastructure systems. By placing digital data in the real world, AR improves situational awareness by enabling operators to identify irregularities and take immediate action in response to new threats. AR applications in CIP can give decision-makers immediate insights into operational status, security warnings, and system health, enabling them to make informed decisions in crisis moments. Moreover, AR-based training simulations can help staff gain practical experience, enhancing their preparedness and response skills. AR presents potential advantages for CIP, particularly in improving situational awareness, facilitating real-time decision-making, and enhancing operator training. However, AR-based solutions require robust data integration from multiple sources, precise calibration, and secure real-time connectivity to prevent cyber vulnerabilities. While industries like defence and manufacturing have demonstrated AR’s practical benefits, large-scale deployment in CIP will depend on the availability of high-fidelity digital twin environments and secure cloud-based AR processing capabilities.

Resilient and adaptive control systems are essential for maintaining operational functionality during disruptions as CNIs become increasingly interconnected and complex. Resilient control systems employ self-healing mechanisms, distributed control algorithms, and autonomous agents to effectively respond to changing conditions and mitigate the impact of cyberattacks and physical threats. Moreover, blockchain is another critical technology enhancing CIP. Blockchain’s immutable and decentralized ledger presents special abilities to protect private information, confirm transactions, and guarantee the reliability of critical services [134]. Stakeholders can develop a strong framework for securing sensitive infrastructure assets by utilizing blockchain technology for identity management, safe data sharing, and tamper-proof documentation of critical operations. Furthermore, smart contracts on blockchain systems can automate and enforce security measures, guaranteeing compliance with legal requirements and enhancing resilience against emerging threats [135].

Another challenge is about building high-quality cybersecurity datasets. It is essential to ensure the data is relevant, diverse, and accurately labeled. First, source data from multiple environments (e.g., network traffic, system logs, malware samples) to cover a broad spectrum of potential threats and behaviors. Ensure that the dataset includes both normal and malicious activities for balanced representation. Data must be cleaned to remove noise and irrelevant entries that could skew the analysis. Properly labeled data with clear, consistent annotations is essential to support effective training of machine learning models. Finally, the implementation of strong privacy measures can protect sensitive information and verify the integrity of the dataset through regular validation. Also datasets tailored to specific needs like IIoT or LLM are essential to be built.

Agentic AI represents a breakthrough in how organizations can protect and manage critical infrastructure. By leveraging autonomous decision-making, real-time collaboration, and adaptive learning, these AI-driven systems overcome the limitations of rule-based CIP approaches. Whether it involves isolating a compromised network segment in seconds or performing predictive maintenance on vital assets, the swift, adaptive, and intelligent capabilities of Agentic AI are pivotal to ensuring reliability and security in an increasingly complex threat landscape. However, maximizing these benefits requires careful governance, continuous oversight, and the integration of ethical and security considerations at every level. By establishing clear boundaries between human controllers and AI agents, maintaining data integrity, and adhering to industry regulations, enterprises can confidently deploy Agentic AI to build more resilient and future-proof infrastructures [136,137].

### Challenges and Real-World Adoption of Emerging Technologies

While emerging technologies such as digital twins, quantum cryptography, and blockchain offer promising solutions for CIP, their real-world adoption remains limited due to high costs, regulatory barriers, and infrastructure challenges. Despite significant industry interest, large-scale deployments face obstacles related to scalability, cybersecurity risks, and operational feasibility. Digital twins have gained traction in manufacturing, aerospace, and smart cities, but their use in critical infrastructures remains limited due to cybersecurity vulnerabilities and high computational demands. Implementing a digital twin framework requires real-time IoT data integration, AI-driven analytics, and significant cloud storage resources, making it costly and complex. Additionally, concerns about data integrity and attack surfaces have slowed adoption in sectors like energy and defense [128]. Quantum cryptography presents a long-term solution for securing critical communications, yet its deployment is restricted to pilot projects in government and finance. The recent finalization of post-quantum cryptographic standards by NIST highlights its potential, but widespread adoption remains hindered by the need for specialized hardware and high deployment costs. Quantum key distribution requires dedicated fiber optic networks or satellite infrastructure, making its implementation impractical for many CNI operators at this stage [129]. Blockchain technology has been explored for data integrity, identity management, and secure transaction logging, yet regulatory uncertainty and high energy consumption limit its real-world applications. While countries like China and the UAE are integrating blockchain for CNI security and identity verification, adoption remains slow in other regions due to scalability concerns and legal compliance issues. Reports indicate that while most of organizations are interested in blockchain for security applications, only a fraction have implemented it at scale [130,138]. These challenges highlight the gap between technological advancements and practical implementation. Overcoming these barriers will require cross-industry collaboration, regulatory clarity, and improvements in cost-effective deployment models.

The future of CIP is dependent on creativity, collaboration, and proactive security strategies. Through the integration of advanced technologies like digital twins, quantum encryption, and augmented reality, stakeholders can fortify critical infrastructure against constantly changing threats. By investing in their adoption as a strategic and progressive step, we can ensure that essential services continue for future generations.

## 10. Conclusions

As our society increasingly depends on networked digital systems, the risk of cyberattacks targeting CNIs continues to grow. CIP against cyberattacks is necessary to maintain the reliability and stability of critical services. As a review paper, this work provides a structured assessment of cybersecurity threats, best practices, and emerging AI-driven solutions for Critical Infrastructure Protection. By analyzing existing literature and regulatory frameworks, we identify key challenges and propose future directions for research and implementation. Our contribution lies in synthesizing current knowledge and outlining opportunities for improvement in AI-driven cybersecurity strategies. This study has explored various challenges and solutions in Critical Infrastructure Protection, covering cybersecurity risks, privacy concerns, and the role of advanced technologies like Generative AI and LLMs. Effective CIP demands a proactive, multidisciplinary approach that integrates technological advancements, regulatory compliance, and strong collaborations. The practical implementation of Generative AI and LLMs in CIP offers several opportunities for enhanced security and automated response mechanisms. These AI-driven models can assist in real-time threat detection, cybersecurity risk assessment, and automated mitigation strategies by analyzing large datasets and identifying anomalies more efficiently than traditional methods. In sectors such as energy, transportation, and healthcare, LLMs can support cybersecurity teams by automating security monitoring and generating predictive insights. However, practical deployment also requires addressing challenges such as model robustness, adversarial resistance, and integration with existing security frameworks. Future work can be focused on developing AI-based security protocols tailored for critical infrastructure environments to maximize effectiveness and ensure long-term reliability. Ensuring the security of critical infrastructure requires significant investment in cybersecurity defences, the strategic use of emerging technologies, and the development of a resilient organizational culture. While this study evaluates LLM performance using multiple benchmarks, future work will focus on real-world deployment scenarios through extensive simulations. Additional experiments, particularly on adversarial robustness and fine-tuning for specific CNI use cases, will further validate the practical applicability of our approach.

## Figures and Tables

**Table 1:** Some significant cyberattacks toward critical national infrastructure sectors between 2022 and 2024.

Month and Year	Attack Type	Critical National Infrastructure Sector	Area
January 2022	Phishing	Government	USA
February 2022	Ransomware	Energy	Belgium, Germany
March 2022	Data Breach	Government	Italy
April 2022	Ransomware	Finance	Costa Rica
May 2022	DDoS	Transport	UK
June 2022	DDoS	Transport	Norway
July 2022	Misinformation	Communications	Ukraine
August 2022	Data Breach	Government	Montenegro
September 2022	Data Breach	Defence	Mexico
October 2022	Ransomware	Communications	Australia
November 2022	DDoS	Government	India
December 2022	DDoS	Government	Vatican City
January 2023	Ransomware	Government	UK
February 2023	Phishing	Government	Italy
March 2023	Cyber Espionage	Civil Nuclear	China
April 2023	Supply Chain Attack	Communications	Global
May 2023	Data Breach	Communications	USA
June 2023	Ransomware	Health	USA
July 2023	DDoS	Government	Trinidad and Tobago
August 2023	DDoS	Finance	Czech Republic
September 2023	Data Theft	Defence	UK
October 2023	Malware Phishing	Defence	South Korea
November 2023	Data Breach	Space	Japan
December 2023	Encryption Attack	Water	Russia
January 2024	Ransomware	Government	Sweden
February 2024	Data Breach	Health	France
March 2024	Data Leak	Defence	Germany
April 2024	Data Breach	Finance	El Salvador
May 2024	Data Breach	Defence	UK

**Table 2:** Comparison of cybersecurity-evaluation benchmarks (part I).

Dataset	Domain	#Questions	Format	Language(s)	Key Features/Notes
**SECURE [32]**	ICS (Industrial Control System) Security	6 datasets	Knowledge extraction, understanding, reasoning	English	Focused on realistic ICS cybersecurity tasks. Evaluates seven state-of-the-art models. Addresses domain-specific strengths and weaknesses of LLMs. Designed to assess LLMs in practical cybersecurity scenarios. Data sourced from industry-standard sources for reliability.
**NetEval [33]**	Networks	5732	Multiple-Choice	Multi-lingual	Covers 5 sub-domains of Network Operations (NetOps). Designed to evaluate commonsense knowledge and inference in NetOps. Multi-lingual evaluation context. Systematic evaluation of 26 publicly available LLMs. GPT-4 achieves near-human performance; LLaMA 2 shows significant potential.
**DebugBench [34]**	Code Debugging	4253 instances	Debugging tasks	C++, Java, Python	4 major bug categories and 18 minor categories. Bugs implanted via GPT-4 with rigorous quality checks. Evaluates LLM debugging ability in zero-shot settings.
**SecQA [35]**	Computer Security	Not specified (Two versions: v1 and v2)	Multiple-Choice	English	Tailored for evaluating LLMs in the domain of computer security. Derived from the “Computer Systems Security: Planning for Success” textbook. Two versions (v1, v2) designed to assess different levels of complexity. GPT-4 used for question generation. Evaluated on various LLMs (e.g., GPT-3.5-Turbo, GPT-4, Llama-2, Vicuna, Mistral, Zephyr) in 0-shot and 5-shot settings. Highlights the varying capabilities of LLMs in understanding security principles. Benchmark dataset for LLM performance in security-related content.
**SecurityEval [36]**	Code Security (Vulnerability)	130 samples	Code-based tasks	English (code contexts)	75 vulnerability types mapped to CWE. Evaluates the security of automated code-generation models. Demonstrated on GitHub Copilot and InCoder.
**CyberMetric [37]**	Cybersecurity	80/500/2000/10,000	Multiple-Choice	English	Broad coverage of cybersecurity topics, including cryptography, reverse engineering, and risk assessment. Generated using GPT-3.5 and Retrieval-Augmented Generation (RAG). Validated by human experts, with over 200 h invested in ensuring accuracy. Evaluated on 25 state-of-the-art LLMs and 30 human participants.
**SecEval [38]**	Cybersecurity	2000+	Multiple-Choice	English	The first benchmark specifically created for evaluating cybersecurity knowledge in Foundation Models. Covers 9 domains: Software Security, Application Security, System Security, Web Security, Cryptography, Memory Safety, Network Security, and PenTest. Questions generated by prompting OpenAI GPT-4 with authoritative sources such as open-licensed textbooks, official documentation, and industry standards. Meets rigorous quality, diversity, and impartiality criteria.
**PythonSecurityEval [39]**	Code Security (Vulnerability)	Large-scale (specific number not disclosed)	Code-based tasks	English, Python	Focuses on real-world applications, including databases, websites, and operating systems. Introduces Feedback-Driven Security Patching (FDSP) for refining vulnerable code. Leverages static code analysis to enhance vulnerability mitigation. Demonstrates empirical improvements of up to 17.6
**OpsEval [40]**	IT Operations (AIOps)	7184 (MC) + 1736 (QA)	Multiple-Choice & QA	English, Chinese	Comprehensive benchmark designed for LLMs in Ops scenarios. Covers tasks like root cause analysis, O&M scripting, and alert summarization. 20% of the data is open-sourced for preliminary evaluation; the remaining 80% kept private to prevent test leakage. Online leaderboard updated in real time for evaluating new LLMs. Includes questions reviewed by domain experts to ensure credibility. Evaluates LLM techniques in areas like model quantification, QA performance, and hallucination handling.

**Table 3:** Comparison of cybersecurity-evaluation benchmarks (part II).

Dataset	Domain	#Data	Format	Language(s)	Key Features/Notes
**EvilInstructCoder [41]**	Adversarial Attacks on Code LLMs	81 samples (0.5% of instruction dataset)	Malicious code injection tasks	English	Adversarial Code Injection Engine to inject malicious snippets into benign code. Evaluates exploitability of CodeLlama, DeepSeek-Coder, StarCoder2 under adversarial scenarios. Demonstrates significant vulnerabilities in instruction-tuned Code LLMs. Poisoning 0.5% of data yields 76–86% Attack Success Rates (ASR@1). Highlights the need for robust defense mechanisms.
**CS-Eval [42]**	Cybersecurity (comprehensive & bilingual)	42 categories	Multiple-question types	English & Chinese	Systematically organized into three cognitive levels: knowledge, ability, and application. Derived from academic research hotspots and practical industrial applications. Demonstrates that certain models outperform GPT-4 in specific subcategories. Extensive evaluation highlights significant improvements in LLMs over time. Publicly accessible benchmark for cybersecurity LLM tasks.
**CyberSecEval [43]**	Code Security & Compliance	Not specified	Code-based & Compliance tasks	English	Comprehensive benchmark for cybersecurity of LLMs used as coding assistants. Evaluates LLMs on insecure code generation and compliance with malicious requests. Automated test case generation and evaluation pipeline. Evaluated 7 models (Llama 2, Code Llama, GPT families). Identifies the tendency of advanced models to generate insecure code. Provides practical insights for refining model security.
**LLMSecEval [44]**	Code Security	150 NL prompts	Natural Language (NL) to code tasks	English	Prompts describe code snippets vulnerable to MITRE’s Top 25 CWE. Each prompt includes a secure implementation example. Enables evaluation of the security of code generated by LLMs from NL descriptions. Facilitates comparative assessment with secure code examples.
**NYU CTF Dataset [45]**	Cybersecurity CTF Challenges	Diverse range (compiled from popular competitions)	Challenge-based tasks	English	Scalable, open-source benchmark for CTF problem-solving. Includes metadata for LLM testing and adaptive learning. Integrates advanced function calling and external tool usage. Fully automated system with enhanced workflow for task evaluation. Evaluates 5 LLMs (both black-box and open-source models). Compares LLM performance to human performance in interactive tasks.
**DIA-Bench [46]**	Mathematics, Cryptography, Cybersecurity, Computer Science	Dynamic (150 templates with mutable parameters)	Text, PDFs, Compiled Binaries, Visual Puzzles, CTF-style Challenges	English	Contains dynamic question templates with mutable parameters. Introduces four new metrics to assess reliability and confidence. Tested on 25 LLMs, highlighting adaptive intelligence. Evaluates models’ adaptive intelligence and confidence across varying tasks. Publicly available on GitHub for reproducibility.
**eyeballvul [47]**	Large-Scale Vulnerability Detection	24,000+ vulnerabilities across 6000+ revisions	Code-based tasks	English	Updated weekly from open-source vulnerabilities. LLM-based scorer compares model output to known vulnerabilities. 55 GB in size; covers 5000+ repositories.
**AttackER dataset [48]**	Cyber-Attack Attribution	Not specified	NER-based (annotated cybersecurity texts)	English	First dataset focusing on extracting attribution information for cyber-attacks. Provides rich annotations, including multi-sentence spans. Highlights contextual details for better understanding of attribution. Demonstrates the potential of LLMs for advanced Named Entity Recognition (NER) in cybersecurity tasks. Designed to support cybersecurity analysts with attacker-oriented countermeasures and legal actions.
**CYBERSECEVAL 3 [49]**	Cybersecurity Risk Measurement	8 distinct risks	Various (e.g., offensive security, social engineering)	English	Expands on prior benchmarks with new offensive security areas. Evaluates Llama 3 and other SOTA models with and without mitigations. Covers automated social engineering, scaling manual offensive cyber operations, and autonomous offensive cyber operations.

**Table 4:** Instruction dataset format.

ine
Below is an instruction that describes a task paired with an input that provides further context. Write a response that appropriately completes the request.
### Instruction:
{instruction}
### Input:
{input}
### Response:
{response}
ine

**Table 5:** Traditional CIP vs. Agentic AI-Enabled CIP.

Dimension	Traditional CIP	Agentic AI–Enabled CIP	Key Benefits
**Monitoring & Anomaly Detection**	Predefined thresholds, manual reviews	Adaptive thresholds via RL, integrates multiple data sources	Real-time detection, fewer false positives, fast zero-day threat identification
**Incident Response**	Manual playbooks, limited automation	Automated workflows, AI-driven isolation/failover	Faster containment, consistent and scalable responses
**Predictive Maintenance**	Schedule-based, siloed data	Data-driven forecasting, early failure detection	Reduced downtime, cost savings, proactive asset management
**Policy & Compliance**	Periodic, manual checks	Real-time validation, automatic non-compliance flags	Continuous compliance, automated reporting, strengthened governance
**Scalability & Flexibility**	Hard to scale, infrastructure-heavy	Modular architecture, easy integration	Minimal overhauls, rapid expansion, adaptable system design
**Cross-Agency Collaboration**	Manual processes, slow info sharing	Multi-agent synchronization, real-time insights	Coordinated responses, streamlined crisis management, enhanced transparency

**Table 6:** Key future directions in CIP and their impact.

Future Direction	CIP Impact
**Digital Twins**	Offers virtual replicas of physical assets for proactive risk mitigation, enhanced operational visibility, and real-time analytics. However, adoption in CNI is slow due to cybersecurity risks, high computational demands, and real-time data synchronization challenges [128].
**Quantum Computing**	Enables advanced threat detection, vulnerability analysis, and optimization in resource allocation; can significantly speed up critical computations.
**Quantum Cryptographic**	Provides quantum-resistant cryptographic techniques to secure data at rest and in transit, protecting CNIs from advanced quantum attacks. However, deployment requires costly infrastructure upgrades (fiber-optic QKD networks, quantum processors), and industry adoption is still limited to pilot programs in finance and defense [129].
**Augmented Reality**	Improves situational awareness for operators; real-time overlays of system status and threat alerts facilitate rapid decision-making.
**Resilient & Adaptive Control Systems**	Uses self-healing and distributed control to maintain operations under stress, mitigating cyberattacks and physical disruptions.
**Blockchain**	Ensures tamper-proof data exchange, secure identity management, and immutable audit trails to bolster trust in critical operations. However, high energy consumption, slow transaction speeds, and regulatory challenges limit large-scale CNI adoption [130].
**High-Quality Cybersecurity Datasets**	Enables robust ML model training; diversified, accurately labeled data improves threat detection and minimizes false positives.
**Agentic AI**	Leverages autonomous decision-making, real-time orchestration, and adaptive learning for proactive, swift, and scalable CIP solutions.

## Data Availability

No new data were created or analyzed in this study. Data sharing is not applicable to this article.

## Abbreviations

CNI	Critical National Infrastructure
DDoS	Distributed Denial-of-Service
APT	Advanced Persistent Threat
ICS	Industrial Control System
IR	Incident Response
BCP	Business Continuity Planning
AI	Artificial Intelligence
CIP	Critical Infrastructure Protection
LLM	Large Language Model
MTTF	Mean Time to Failure
MTTR	Mean Time to Restore, Respond, or Repair
NetOps	Network Operations
CWE	Common Weakness Enumeration
FDSP	Feedback-Driven Security Patching
CTF	Capture the Flag
DIA	Dynamic Intelligence Assessment
NER	Named Entity Recognition
GDPR	General Data Protection Regulation
HIPAA	Health Insurance Portability and Accountability Act
PDPA	Personal Data Protection Act
SSCA	Safety and Security Co-analysis
MTTA	Mean Time to Attack
SMPC	Secure Multi-Party Computation
RLHF	Reinforcement Learning from Human Feedback
NIS	Network and Information System
AR	Augmented Reality
RAG	Retrieval-Augmented Generation
